# Visuomotor Learning Generalizes Around the Intended Movement^1^,^2^,^3^

**DOI:** 10.1523/ENEURO.0005-16.2016

**Published:** 2016-04-29

**Authors:** Kevin A. Day, Ryan T. Roemmich, Jordan A. Taylor, Amy J. Bastian

**Affiliations:** 1 Department of Biomedical Engineering, The Johns Hopkins University School of Medicine, Baltimore, Maryland 21205; 2 Motion Analysis Laboratory, Kennedy Krieger Institute, Baltimore, Maryland 21205; 3 Department of Neuroscience, The Johns Hopkins University School of Medicine, Baltimore, Maryland 21205; 4 Department of Psychology, Princeton University, Princeton, New Jersey 08540

**Keywords:** adaptation, generalization, motor learning, reaching, upper extremity, visuomotor

## Abstract

Human motor learning is useful if it generalizes beyond the trained task. Here, we introduce a new idea about how human visuomotor learning generalizes. We show that learned reaching movements generalize around where a person intends to move (i.e., aiming direction) as opposed to where they actually move. We used a visual rotation paradigm that allowed us to disentangle whether generalization is centered on where people aim to move, where they actually move, or where visual feedback indicates they moved. Participants reached to a visual target with their arm occluded from view. The cursor feedback was rotated relative to the position of their unseen hand to induce learning. Participants verbally reported their aiming direction, reached, and then were shown the outcome. We periodically introduced single catch trials with no feedback to measure learning. Results showed that learning was maximal at the participants’ aiming location, and not at the actual hand position or where the cursor was displayed. This demonstrates that visuomotor learning generalizes around where we intend to move rather than where we actually move, and thus introduces a new role for cognitive processes beyond simply reducing movement error.

## Significance Statement

Generalization is an important feature of human motor learning, as movements learned within a specific environment often apply broadly to unfamiliar environments without further training. Previous work has produced mixed, and often conflicting, results when studying how the nervous system generalizes learned movement patterns. Here, we provide a new perspective on motor learning generalization of visuomotor rotations that reconciles many prior conflicting findings by showing that learned motor patterns generalize around where we intend to move rather than where we actually move. These findings indicate that new movements are learned around a cognitive representation of the movement, and demonstrate a new role for cognitive contributions to motor learning.

## Introduction

Human motor control is impressively flexible; movements learned and practiced within a specific context often generalize to novel situations (Poggio and Bizzi, 2004). Consider, for example, learning to play golf—while it is useful to learn to swing a single club appropriately, we ultimately aim to use the entire set. Fortunately, learning to swing one club often improves our ability to swing others, especially those very similar to the club that has been well practiced (Lam et al., 2010).

Complex actions like swinging a golf club are learned through interacting mechanisms, some more explicit and some more implicit. Here we studied how recently learned movements generalize when they are acquired using a combination of explicit and implicit learning mechanisms. These mechanisms are often studied using a visuomotor reaching task where participants reach for a visual target while a cursor represents the position of their hand (Mazzoni and Krakauer, 2006; Taylor and Ivry, 2011; Taylor et al., 2014). After some practice, a perturbation is applied that rotates the cursor position away from the hand position. Implicit learning changes the relationship between the hand position and expected cursor location involuntarily, and is thought to be driven by sensory prediction errors (i.e., any discrepancy between the expected and actual location of the cursor; Tseng et al., 2007). Explicit learning is characterized by a conscious change in the aim of the reach and is driven by target error (i.e., any discrepancy between the actual locations of the cursor and the target; Mazzoni and Krakauer, 2006). Both implicit and explicit learning change how we move, but only explicit learning changes how we think we move. Therefore, an important question remains: does motor learning generalize around how we move or how we think we move?

It has been difficult to answer this question in previous studies because participants feel that their hand is moving with the cursor by the end of adaptation, although by this point it is in fact moving in a rotated direction (Izawa and Shadmehr, 2011). This makes previous work showing that learning generalizes narrowly around the target (Ghilardi et al., 1995; Krakauer et al., 2000; Fernandes et al., 2014) difficult to interpret, as participants perceive that their hand is moving directly to the target. Using a paradigm where aiming landmarks are provided during learning, participants have knowledge that their hand is rotated relative to the cursor (Taylor and Ivry, 2011), and thus willingly aim their reaches away from the target to counteract the perturbation (Taylor et al., 2014). We used this paradigm to dissociate whether learning generalizes around the perceived hand position (i.e., aiming) or target position.

Here we show that, in a situation where participants are asked to report their aiming location, implicit motor learning generalizes around the aiming location (i.e., where they intended to move) rather than the target location (i.e., where the cursor moved). We directly measured implicit learning within the task, whereas most previous work has inferred it by subtracting the aim from the reaching angle (Taylor et al., 2014). Across experiments, we tested implicit learning at different points in the workspace, some closer to the aiming location and some closer to the target. We found that implicit learning generalizes maximally at the location where participants most frequently aimed their reach, suggesting that humans generalize visuomotor learning around a cognitive construct of how we think we move rather than how we actually move.

## Materials and Methods

### Participants

Seventy young, healthy adults were recruited for this experiment (18 male, 52 female; mean ± SD age, 25 ± 5 years). All participants provided written, informed consent prior to taking part in the experiment. The experimental protocol was approved by The Johns Hopkins Medicine Institutional Review Board. All participants were right handed, as confirmed by the Edinburgh handedness inventory, and were free of any neurological and musculoskeletal conditions.

### Protocol

Participants sat facing a computer monitor (47 × 30 cm) oriented in the horizontal plane at eye level. The monitor was positioned 30 cm above a digitized touchpad such that the monitor obscured visual feedback of the participant’s hand as he/she used a stylus (Intuous 3, Wacom) to perform center-out reaching trajectories. Visual feedback of hand trajectories was provided on the monitor by a circular cursor (3.5 mm in diameter).

**Figure 1.**
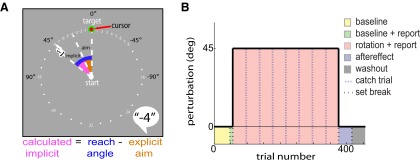
Experimental design. ***A***, General setup and convention for aiming landmarks and target. Participants were provided with a ring of numbered landmarks—spaced 5.625° apart—to report their aim prior to each reach. The target was always presented directly ahead of the starting location at 0°. The calculation of the implicit component of adaptation was performed by subtracting the participant’s self-reported explicit aim from the reach angle for each trial. ***B***, Experimental protocol. In the baseline block (yellow), participants completed 48 trials with veridical end point feedback. In baseline plus report (green), participants practiced reporting their aim while receiving veridical end point feedback for eight trials. In the rotation plus report, participants were introduced to a 45° CW visuomotor rotation while still verbally reporting their aiming location for 320 trials. In the aftereffect block (purple), participants were instructed to reach directly for the target in the absence of both aiming landmarks and end point feedback for 40 trials. The rotation was removed from these trials to measure the amount of sensorimotor recalibration present. Last, the washout block (gray) restored veridical end point feedback for 40 trials. No-feedback, no-rotation single catch trials (purple dashed lines) were periodically collected every 40 trials during the rotation block. These trials had the same format as the aftereffect block (purple).

Trials began with the participant moving his/her hand within a starting circle (5 mm in diameter) in the center of the screen. Prior to positioning the cursor inside the starting circle, participants were provided with information only about the radial distance of their hand from the center to prevent them from gaining additional information about the mapping between hand and cursor position. After maintaining the cursor position within the starting circle for 1 s, a green target circle (7 mm in diameter) was displayed 70 mm from the center and directly ahead of the starting location (0° location). Participants were instructed that the goal of the task was to pass their cursor through the green target by making a fast and accurate reach. All participants were given end point feedback about reach direction in the form of a stationary red circular cursor (3.5 mm in diameter) after the reach exceeded the 70 mm target distance (Fig. 1*A*). If the red cursor was within the diameter of the green target circle, a “ding” sound was played, indicating a successful trial (i.e., the target had been hit). Otherwise, a “buzz” sound was played, indicating that the reach missed the target. If the participant did not make the reach within 500 ms, a “too slow” auditory cue was prompted.

Figure 1*B* shows the experimental paradigm. First, participants completed 48 baseline trials with veridical end point feedback (yellow block). Second, they reached for eight trials with veridical end point feedback and were instructed to report the visual landmark toward which they aimed each reach (green block). This verbally reported landmark served as our measure of the explicit component of learning (Fig. 1*A*). The consecutively numbered landmarks were evenly distributed 5.625° apart at the same radius as the target. The green target was located at the “0” landmark for all learning trials throughout the entire experiment (Fig. 1*A*). Participants then reached for 320 trials with a 45° clockwise (CW) visuomotor rotation (pink block). Within this block, participants were instructed to continue to report their aiming landmark as they attempted to hit the green target with their red cursor. After the rotation block, the end point feedback and aiming landmarks were removed for 40 trials to measure the aftereffect (purple block). Participants were instructed to aim directly for the green target and were given a neutral “knock” sound when their movement magnitude exceeded 70 mm and the trial was over. Finally, participants performed 40 washout trials in which veridical end point feedback returned, and they were instructed to aim directly at the green target (gray block). The participants were not required to report their intended aiming direction in the last two blocks.

Importantly, here we also included a direct measurement of implicit learning by collecting “catch trials.” In past studies, the implicit component of visuomotor adaptation was abstracted by subtracting the reported aim from the reach angle (Taylor et al., 2014)—referred to herein as “calculated implicit.” This is illustrated in Figure 1*A*. Here, we assayed the participants’ reported aiming locations but also collected single catch trials every 40 trials during the rotation block to directly measure implicit learning. These single catch trials had the same format as the aftereffect measurement trials (purple block) in that end point feedback and landmarks were removed, and participants were instructed to reach to the target. As is common in aftereffect measurement (Mazzoni and Krakauer, 2006; Taylor and Ivry, 2011; Taylor et al., 2014), instructing the participants to aim directly at the target allows us to control the explicit component of learning and therefore directly probe the implicit component. Changing where we presented these catch trials relative to the trained target location allowed us to characterize how implicit learning generalized throughout the workspace. Below, we step through our reasoning for selecting various locations throughout the workspace to collect these catch trial and aftereffect measurements.

**Figure 2.**
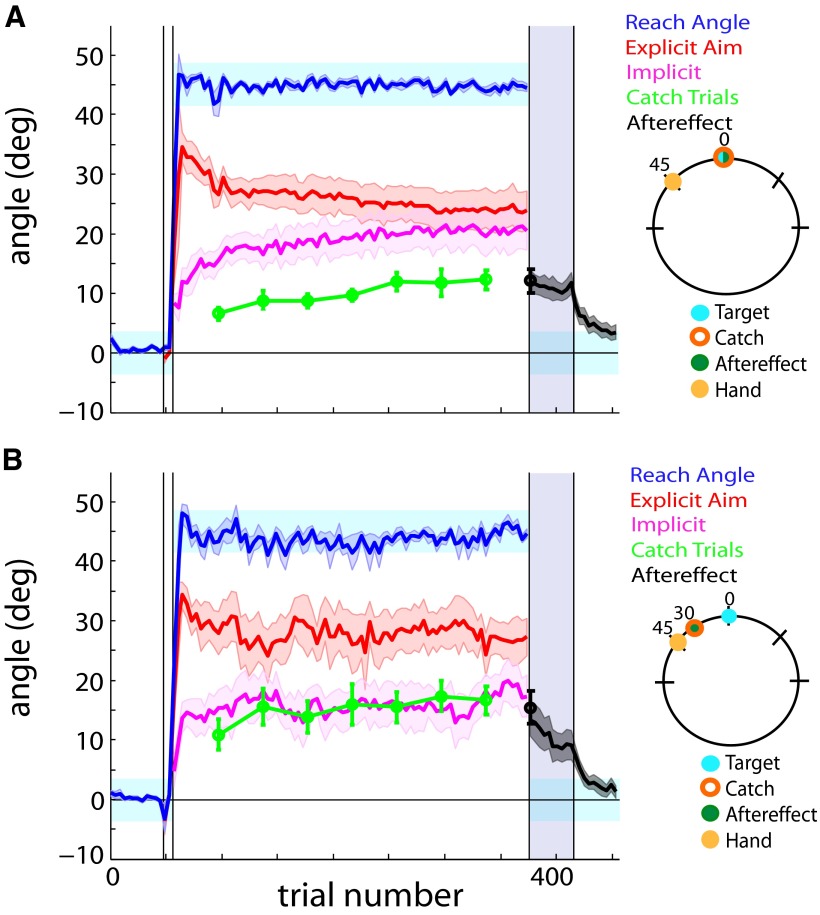
Target and aim groups. The catch trials (green) and aftereffect (black) were measured at various locations in the workspace relative to the 0° target location (key to the right illustrates catch and aftereffect locations relative to the target). Implicit aim (magenta) was calculated from subtracting explicit aim (red) from reach angle (blue). The light blue shaded areas represent the target area. The purple shaded region during the aftereffect (trials 377-417) denotes no-feedback trials. The black error bar (trial 377) displays the magnitude of the first aftereffect trial. Error bars and shaded error regions denote the SEM. ***A***, Target group. The catch trials (green) and aftereffect (black) were measured at the 0° trained target location. ***B***, Aim group. The catch trials (green) and aftereffect (black) were measured 30° CCW of the target location, corresponding to the most frequently reported aim location. Moving the catch trial/aftereffect location to the 30° CCW aiming location removes the offset between the calculated implicit (magenta) and catch trial measurements (green) when collected at the target location observed in ***A***.

We first tested a group of participants that was instructed to aim at the trained target (i.e., 0° location) for all catch and aftereffect trials (called the “target group” here). We noticed an interesting discrepancy between the calculated implicit component and the directly measured catch trial magnitudes (Fig. 2*A*, see the difference between pink and green curves). Given that adaptation is thought to generalize maximally around the target location (Krakauer et al., 2000), why did the calculated implicit component and catch trial magnitudes, when tested at 0°, not match?

We developed two competing hypotheses to answer this question. First, it is possible that the calculated implicit component consistently overestimates the true implicit learning as measured by our catch trials. Alternatively, the calculated implicit component and catch trial magnitudes at 0° may differ because learning generalizes around the aiming location rather than the target location. For example, consider a participant who aimed at 28° (i.e., approximately the “−5” landmark) throughout the majority of the rotation block while the hand actually moved to a 45° location (i.e. the “−8” landmark). When the participant is instructed to change their aim to 0° during the catch trials, our aiming generalization hypothesis predicts that the catch trial magnitude should be reduced compared with the calculated implicit component. This hypothesis then also predicts that the difference between the calculated implicit component, and catch trial magnitudes should depend on the location of the catch trials within the workspace.

Accordingly, we tested several groups to probe the catch trials and aftereffect at different locations. The correspondence between these direct measures of implicit learning and our calculated implicit learning will form the basis for our characterization of the generalization of visuomotor adaptation. We tested one group with catch and aftereffect trials at a location close to the approximate mean reported aiming location among all of the participants, or 30° counterclockwise (CCW) of the target (“aim” group). Additionally, we collected two groups where the catch trials and aftereffects were collected in locations in opposite directions of the rotation where participants had neither reached nor aimed throughout the experiment (“extreme CCW” and “extreme CW” groups). The extreme CCW group was collected at 60° CCW of the target, while the extreme CW group was collected at 90° CW of the target. To explore the effect of cursor location on generalization, we tested a group at a 30° CW location where the participants had never reached their hand or aimed in the location of the catch trials, yet had seen the cursor land in that position (“cursor” group). To explore the effect of reach location on generalization, we tested a group in which the catch and aftereffect trials were positioned at the 45° CCW location relative to the target where participants most frequently reached during the rotation block (“hand” group). Further, we tested a group with the catch trials at the aiming location (30° CCW) and the aftereffect at the target location (0°) to determine whether the generalization effect can be observed within participants (“aim/target” group). Each group consisted of 10 participants.

### Data analysis

Reach angle was calculated as the hand angle when the movement trajectory crossed the 70 mm target distance relative to the starting position. A positive reach angle indicated that the participant reached CCW of the target. Over the course of the rotation block, participants corrected for the angular error between the cursor and target by adjusting their reach angle in the CCW direction. The optimal compensation for the perturbation was a 45° reach angle. We calculated the aiming angle by multiplying the participants’ reported landmark numbers by −5.625° (negative to maintain the sign convention). The calculated implicit was obtained by subtracting the explicit aiming angle from the reach angle (Fig. 1*A*; Taylor et al., 2014). Reach angle, aiming angle, and calculated implicit were all binned (*n* = 4 trials/bin) for analysis and display. Plateau values for each of these measures were calculated by averaging the last 100 trials of the rotation block.

To quantify the extent to which the measured catch trials matched the calculated implicit component, we calculated the proportion (φ) of catch trial *i* to the calculated implicit bin collected prior to that catch trial, as follows:

$$\varphi_{i}=\frac{catch\;trial\;magnitude_{i}}{mean{\left(calculated\;implicit\right)}_{i-4:i-1}}$$

To eliminate outliers but still maintain 90% of the data (441 of 490 values), we eliminated φ values greater than 2.3 or less than −2.3, respectively. A one-way ANOVA with catch trial number as a factor revealed no difference in φ values across catch trials (*F*_(6,434)_ =0.41, *p* = 0.87). This suggested that the discrepancy between the calculated implicit component and catch trial magnitudes did not change as a function of trial number. Thus, we averaged these proportions across the seven collected catch trials for each subject. Because the aim group and aim/target group experienced identical paradigms until the start of aftereffect measurement, we combined the data from the two groups for analysis of the proportion φ. Therefore, the 30° catch trial data contain 20 participants, while data for all other catch trial locations are composed of 10 participants. To test whether an offset was present in each group, we ran one-sample *t* tests with a null hypothesis of proportion φ equal to 1 (i.e., perfect generalization). The fitted distributions and corresponding *R* ^2^ values for the cursor, aim, and reach angle during the rotation block (see Fig. 5, green, red, and gold curves) were obtained using the curve-fitting toolbox in MATLAB (MathWorks). Further, we used a one-way ANOVA to observe the main group effect on the proportion φ. *Post hoc* analysis to compare individual groups was performed using Fisher’s LSD method. Our aftereffect analysis was performed using a mixed-design repeated-measures ANOVA with time and group as factors.

For our within-subject analysis of generalization, we built generalization curves for each of the 70 participants. We calculated the mean magnitude of implicit learning at every aiming location for each subject. This allowed us to observe the generalization of implicit learning as participants aimed farther away from their most frequently reported aiming location during the rotation block. We only included aiming locations in our analysis where data were available for >10 of our 70 subjects. Because participants did not report aiming at all aiming locations (particularly those far away from their most frequently reported aim), this inclusion criterion confined our analysis to 40° CCW and 35° CW of the most frequent aiming location. We used the *fminspleas* function within MATLAB (MathWorks) to fit a cosine function to our within-subject generalization data. We then performed a correlational analysis of the generalization of implicit learning and the absolute angle between the most frequent aim and the actual aim. We set our α level at 0.05 for all statistical analyses.

## Results

### Catch trial analysis

In this experiment, we sought to discern whether the participants learned around where they thought their hand was going, where their hand was actually going, or where visual feedback was displayed during a visuomotor rotation task. All participants successfully reached toward the target at baseline, and there was no group effect on reach angle during baseline (one-way ANOVA, *F*_(5,64)_ = 0.46, *p* = 0.81) or on reported aim during baseline plus report (*F*_(5,64)_ = 1.51, *p* = 0.20). Similarly, we did not observe any group effects on reach angle (one-way ANOVA, *F*_(5,64)_ = 1.45, *p* = 0.22) or reported aim (one-way ANOVA, *F*_(5,64)_ = 0.33, *p* = 0.90) at plateau during the rotation block, indicating that all participants were able to successfully achieve the goal of hitting the green target.

At plateau, participants in the target group consistently hit the target with a mean (±SE) reach angle of 45.04 ± 0.28° while reporting an aim of 23.56 ± 3.12° (Fig. 2*A*). As mentioned in Materials and Methods, we observed an offset between the measured catch trials and the calculated implicit component, as the calculated implicit consistently overestimated the measured catch trial magnitudes. Participants within this group showed mean φ values of 0.64 ± 0.13 (one-sample *t* test, *t*_(9)_ = −2.78, *p* = 0.02). Previous literature suggests that a portion of implicit learning decays over short time scales (Miyamoto et al., 2014). However, further analysis of the intertrial interval (ITI) prior to the collection of the catch trials and ITI between normal rotation trials revealed that, regardless of whether the next trial is a catch trial or a rotation trial, the ITI was similar (paired *t* test, *t*_(6)_ = −0.30, *p* = 0.78). Thus, the observed offset cannot be reconciled by the notion that a greater ITI prior to catch trials leads to a greater decay of implicit learning. Additionally, we observed no difference between the magnitude of the last catch trial (12.26 ± 1.53°) and first trial of the aftereffect measurement (12.09 ± 1.97°; paired *t* test, *t*_(9)_ = 0.09, *p* = 0.93).

These results prompted us to explore whether the discrepancy between the measured and calculated implicit component could be due to incomplete generalization around the target. As such, we altered the paradigm so that the catch trial and aftereffects were collected 30° CCW of the target location (Fig. 2*B*). This location was the approximate average of the reported aiming location of all participants in the study. In this aim group, the reported aim and reach angles plateaued at 27.38 ± 3.13° and 45.32 ± 0.30°, respectively. The offset between the measured and calculated implicit components that was observed in the target group was no longer present in the aim group. The mean φ values for this group were 0.92 ± 0.11 (one-sample *t* test, *t*_(9)_ = −0.70, *p* = 0.50). When combined with the aim/target group (discussed further below), the mean cumulative φ values for the catch trials collected at 30° were 0.97 ± 0.09°, indicating that there is no significant difference between measured and calculated implicit learning (one-sample *t* test, *t*_(19)_ = −0.35, *p* = 0.73), and near-perfect generalization.

**Figure 3.**
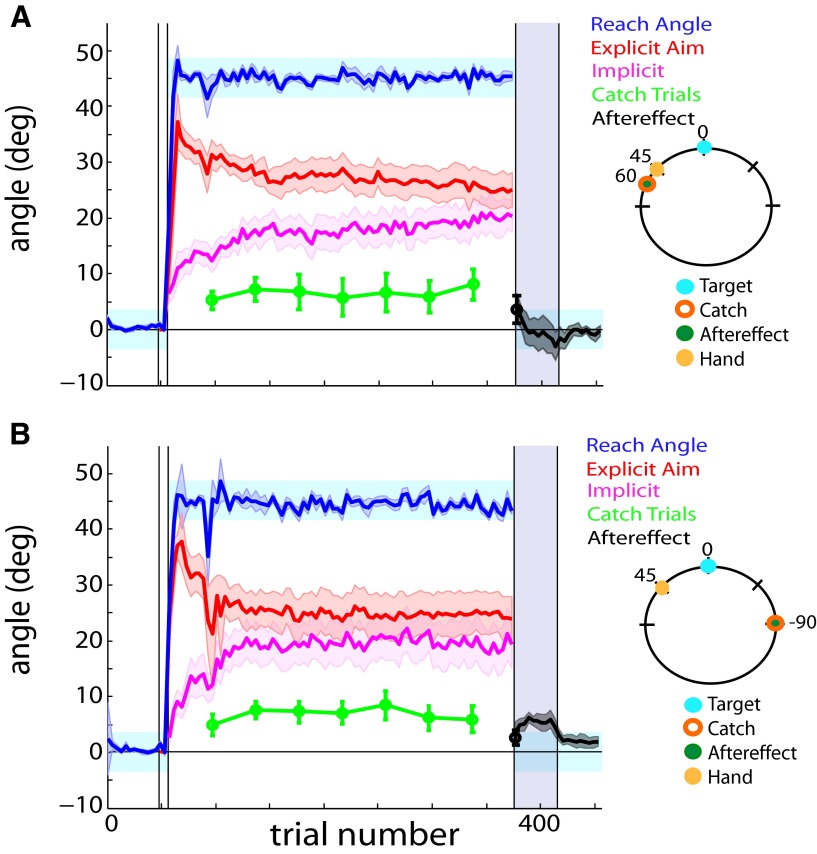
Extreme CCW and extreme CW groups. The trained target remained at the 0° location, while the catch trials and aftereffect were probed in untrained regions of the workspace. ***A***, Extreme CCW group. The catch trials (green) and aftereffect (black) were measured 60° CW of the target location. ***B***, Extreme CCW group. The catch trials (green) and aftereffect (black) were measured 90° CW of the target location. Error bars and shaded error regions denote the SEM.

The hallmark of generalization is that locations farther away from the trained location show diminished learning (Krakauer et al., 2000). If implicit learning generalizes around the aiming location (i.e., 30° CCW of target), we would expect that the degree to which the catch trial magnitudes match the calculated implicit component will decrease as we probe catch trial locations farther from where the participants concentrated their aim. We tested the extremes of the pattern of generalization by testing extreme CCW and extreme CW groups at locations where participants had neither aimed nor reached throughout the rotation block—60° CCW and 90° CW, respectively. By the end of the rotation block, participants in the extreme CCW group aimed at 25.32 ± 2.90° while reaching toward 44.71 ± 0.38° (Fig. 3*A*). The measured catch trial magnitudes decreased markedly as the mean φ values were 0.27 ± 0.15 (one-sample *t* test, *t*_(9)_ = −4.78, *p* = 0.001). These results suggest that, while these participants showed implicit learning similar to that of the aim group (∼15-20° at plateau), learning did not generalize to locations in the workspace where they had never aimed or reached. To examine whether this finding extends to portions of the workspace in the direction of the perturbation, we tested an extreme CW group. At plateau, these participants aimed at 24.23 ± 3.73° while reaching toward 45.53 ± 0.31° (Fig. 3*B*). Similar to the extreme CCW group, we observed a significant offset in the extreme CW condition with mean φ values of 0.50 ± 0.16 (one-sample *t* test, *t*_(9)_ = −3.16, *p* = 0.012).

**Figure 4.**
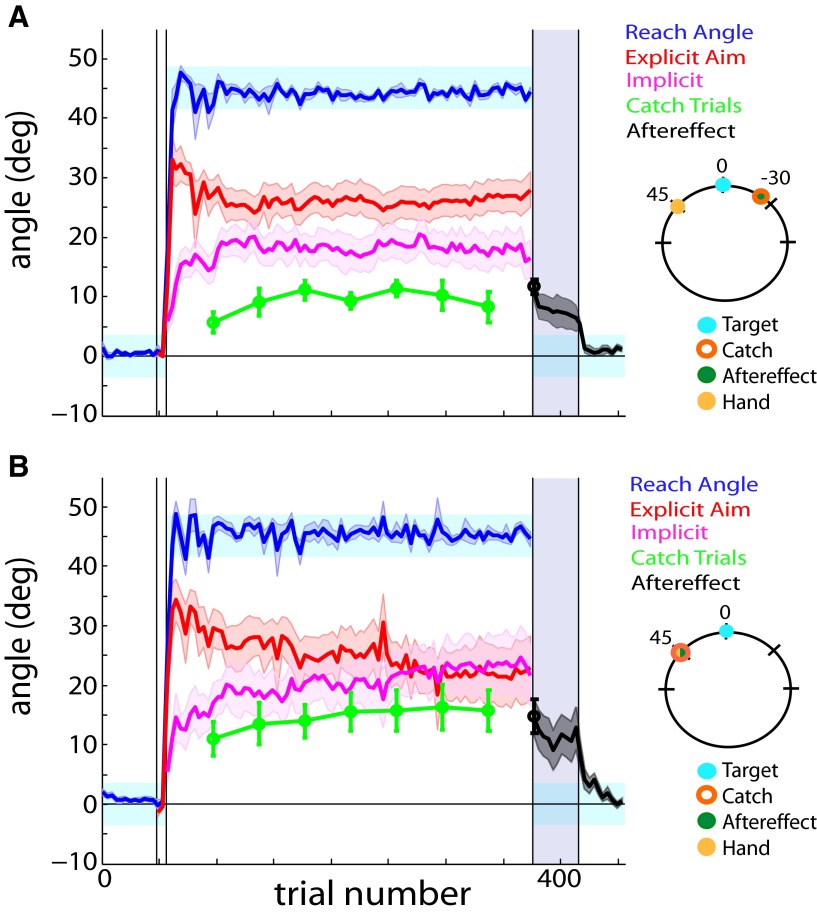
Cursor and hand groups. The trained target remained at the 0° location, while the catch trials and aftereffect were probed in untrained regions of the workspace. ***A***, Cursor group. The catch trials (green) and aftereffect (black) were measured 30° CW of the target location. ***B***, Hand group. The catch trials (green) and aftereffect (black) were measured 45° CCW of the target location, corresponding to the location where the participants reached their hands most frequently during the rotation block. Error bars and shaded error regions denote the SEM.

Perhaps end point feedback of the cursor influenced the extent to which implicit learning generalizes within the workspace. Accordingly, we tested a cursor group at a location where the participants have never reached or aimed during the experiment, yet had seen the cursor land in that location (30° CW; Fig. 4*A*). Aiming and reaching location plateaued at 25.85 ± 2.57° and 45.34 ± 0.24°, respectively. The calculated implicit component significantly overshot the measured catch trials with mean φ values of 0.49 ± 0.06 (one-sample *t* test, *t*_(9)_ = −8.36, *p* < 0.001).

Because the participants’ hand movements were centered around 45° during a vast majority of the rotation block, we collected a hand group to observe whether the pattern of generalization was biased by the position of the repeated motion (45° CCW; Fig. 4*B*). The aiming and reaching angle plateaued at 21.48 ± 4.38° and 44.36 ± 0.54°, respectively. Notably, the observed offset between calculated and measured implicit learning persisted with a mean φ value of 0.66 ± 0.08 (one-sample *t* test, *t*_(9)_ = −4.42, *p* = 0.002). This result suggests that implicit learning generalizes incompletely around the repeated reach location, hinting that we may learn around a cognitive construct of where we think we are aiming rather than where our hand actually moves during a visuomotor rotation task.

**Figure 5.**
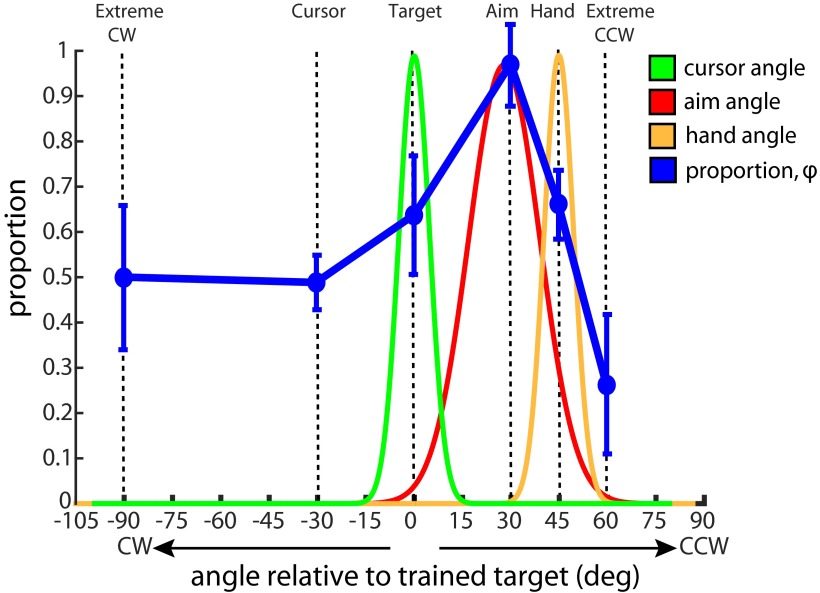
Implicit adaptation generalizes around the aiming location (30° CCW). φ denotes generalization, calculated as the proportion of the catch trial magnitudes (Figs. 2-4, green error bars) to the calculated implicit (Figures 2-4, magenta trace). When plotted as a function of angle relative to the trained target location, the φ values reveal a generalization curve that is centered around the aiming location (blue). Error bars denote the SEM. This is overlaid on fitted Gaussian distributions for the cursor (green; *R* ^2^ = 0.996), reported aim (red; *R* ^2^ = 0.969), and hand (gold; *R* ^2^ = 0.996) angles for all 70 participants during the rotation block. The distributions are normalized to their respective means.

Using φ as our primary outcome measure, we can further validate the pattern of generalization observed during this visuomotor rotation task (Fig. 5). A one-way ANOVA using catch trial location as a factor revealed a significant difference in φ values across conditions (one-way ANOVA, *F*_(5,64)_ =5.23, *p* = 0.0004). *Post hoc* multiple comparison analysis using Fisher’s LSD test revealed that the measured catch trials in the 30° CCW catch trial conditions (aim and aim/target combined) matched the calculated implicit to a significantly greater extent than did the extreme CW (*p* = 0.003), cursor (*p* = 0.002), target (*p* = 0.033), hand (*p* = 0.047), and extreme CCW (*p* ≤ 0.001) conditions. A one-way ANOVA confirmed that the ITI prior to the collection of catch trials remained consistent across all conditions (one-way ANOVA, *F*_(5,64)_ = 0.69, *p* = 0.63), thus eliminating the concern that implicit learning may have temporally decayed to a greater degree for one group than for others. Therefore, participants appear to learn maximally around their most frequent aiming location. At this location, independently measured catch trial findings are consistent with the implicit component calculated from the reach angle and reported explicit aim.

### Aftereffect analysis

**Figure 6.**
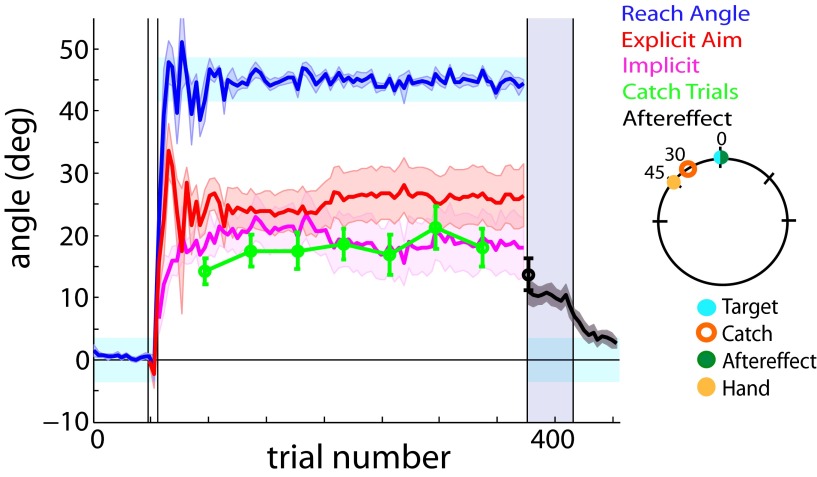
Mixed aim/target group to explore the within-subject generalization effect. The catch trials (green) were measured 30° CCW of the target location, which is similar to that of the aim group in Figure 2*B*, corresponding to the location where the participants reached their hands most frequently during the rotation block. The aftereffect trials (black) were measured at the target location. Note that there is a drop from the catch to the aftereffect trials; this is because the catch trials were performed at the aiming location, and aftereffect trials at the target location. Error bars and shaded error regions denote the SEM.

In the fourth block of the experiment, we measured the participants’ aftereffects by removing the cursor feedback, visuomotor rotation, and landmarks, and instructing the participants to aim directly for the green target. The aftereffect is a measure of the participants’ sensorimotor recalibration and arises from an updating of an internal model (Shadmehr and Mussa-Ivaldi 1994; Wolpert and Kawato, 1998; Taylor et al., 2014). Indeed, the aftereffect remains the gold standard for evaluating the degree of sensorimotor recalibration that results from a visuomotor adaptation task. We used a mixed-design repeated-measures ANOVA to evaluate how the degree of implicit learning varied between catch trial measurements at plateau (last two catch trials) and early aftereffect measurement (first four trials) in addition to the group effects on the difference between the two measures. There were main effects of time (mixed design, repeated-measures ANOVA, *F*_(1,63)_ = 15.11, *p* < 0.001) and group (mixed design, repeated-measures ANOVA, *F*_(6,63)_ = 4.20, *p* = 0.001), as well as a significant interaction (mixed design, repeated-measures ANOVA, *F*_(6,63)_ = 3.85, *p* = 0.002). *Post hoc* analysis revealed a significant pairwise difference between the catch trial magnitude at plateau and early aftereffect in the aim/target condition (*p* < 0.001; Fig. 6); otherwise, the remaining conditions did not display a difference between catch trial and aftereffect magnitude. Interestingly, these results suggest that the generalization effect we observed across conditions also can be observed within participants because we observe a significant within-subject difference in the degree of sensorimotor recalibration as we probe different locations in the workspace.

### Within-subject analysis

**Figure 7.**
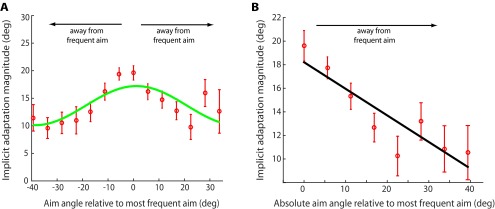
Within-subject generalization. For each participant, the mean implicit learning magnitude was calculated at each reported aiming location. Aiming locations that had data available for >10 of 70 subjects were included. ***A***, Implicit learning magnitude for all 70 subjects. Data for individual subjects were centered on their most frequently reported aiming locations. Thus, 0° here denotes the aiming location that the subject reported most frequently during the rotation block. The green curve denotes a best-fit cosine function. ***B***, Correlational analysis revealed a negative correlation (correlation coefficient = −0.89, *p* = 0.0029) between the absolute angle away from the most frequent aiming location and the generalization of implicit learning. This collapsed all negative and positive aim angles shown in ***A***. As participants reached farther from their most frequently reported aim, the generalization of implicit learning decays. The black trace is a best-fit linear regression (*R* ^2^ = 0.796). Error bars denote the SEM.

To further characterize the within-subject generalization, we built generalization curves for each of the 70 participants across all groups. Figure 7 reveals that, as participants aimed farther from their most frequently reported aiming location, the magnitude of implicit learning decreased. Thus, implicit learning generalized maximally at each individual’s most frequent aiming location and decays as a function of angle away from that aiming location. The best-fit cosine curve to our within-subject generalization data was centered at 1.16°. This phase shift confirms that the generalization of implicit learning was centered on the most frequent aiming location (Fig. 7*A*, 0°). To quantify this relationship, we used a correlational analysis that showed significant correlation between implicit learning magnitude and the absolute value of the angle away from the most frequent aiming location (Fig. 7*B*; correlation coefficient = −0.89, *p* = 0.0029).

## Discussion

Generalization is a critical feature of motor adaptation, as movement patterns adapted within a specific context can carry over more broadly to novel situations. However, how the nervous system generalizes newly adapted movement patterns from trained to untrained environments has been a puzzling question due to mixed results in the literature. Generalization has been thought to be maximal at the trained target location (Ghilardi et al., 1995; Krakauer et al., 2000; Brayanov et al., 2012; Fernandes et al., 2014), the movement direction (Gonzalez Castro et al., 2011), and the planned motor output (Hirashima and Nozaki, 2012), among others. Here we show that visuomotor adaptation generalizes around a cognitive construct of how we think we move, not around characteristics of the movement or learning environment, as previously thought. Specifically, we found that visuomotor adaptation generalized maximally at the location where participants most frequently aimed their reaches—not the target location or the location where they most frequently reached. Interestingly, this phenomenon holds both between and within subjects. These findings provide new evidence for an important role of cognitive processes in the generalization of visuomotor adaptation.

Research has recently begun to explore the influence of explicit cognitive processes on motor adaptation, a motor learning process long thought to be largely cerebellar mediated. To quantify explicit contributions to motor adaptation, previous work assayed the self-reported reaching aim from participants while they adapted to a visuomotor rotation. In that work, the authors assumed that implicit learning was represented by any change in cursor position unaccompanied by a similar change in the explicitly reported aim (Taylor et al., 2014). Here, we implemented a more direct measurement of implicit learning by interspersing no-feedback catch trials throughout the adaptation block. In our study, as expected, all participants showed monotonic implicit learning as calculated by subtracting their reported aim from their reaching angle during adaptation (Taylor et al., 2014). However, the implicit learning measured directly in the catch and aftereffect trials consistently undershot the calculated implicit learning in nearly all testing locations. Only when the catch and aftereffect trials were located precisely at the participants’ aiming locations did our direct measurements of implicit learning align with the indirect measurement used previously. Accordingly, we posit that these results indicate a striking generalization pattern that is centered around the participants’ intended reaching trajectories (i.e., aim).

These findings provide a unifying framework to reconcile mixed behavioral findings from previous work. For example, a previous study found that adaptation generalizes around the target location (Krakauer et al., 2000). However, we suggest that what appeared to be generalization around the target actually may have been generalization around the aiming location. We think this because participants often report that they are reaching directly to the target by the end of the adaptation block when only the target is present (Izawa and Shadmehr, 2011). Thus, it seems likely that the target and aiming locations overlapped in the study by Krakauer et al. (2000), making it difficult to dissociate them. Our findings also clarify the results of other studies reporting that adaptation generalizes at a location other than the target (Heuer and Hegele, 2008, 2011). For instance, Heuer and Hegele (2008) observed that adaptation generalized around a location 45° counterclockwise of the target direction following a 75° clockwise rotation. Interestingly, this 45° shift in the generalization pattern corresponded to the approximate magnitude of the explicit learning shift that the authors observed. Thus, this explicit aim, albeit collected in a slightly different manner from the current study, appears to be the center of generalization, an observation that is consistent with our results. Recent work (Gonzalez Castro et al., 2011) has suggested that motor adaptation is motion referenced rather than plan referenced. While this previous work used force-field learning to dissociate actual and planned movement, there was no dissociation between actual and intended movement (i.e., aim). In Gonzalez Castro et al. (2011), it was assumed that the participants’ plan was to move directly to the target, but recent studies have shown that participants do not aim directly to the target during learning (Taylor et al., 2014; Bond and Taylor, 2015; McDougle et al., 2015; Morehead et al., 2015). If participants were aiming in a direction away from the target, then the unmeasured aim direction and the actual motion direction may have been overlapping, leading them to the belief that a motion-based reference frame was more consistent with the generalization pattern. It is important to note, however, that the neural mechanisms underlying adaptation to a dynamic perturbation differ from those underlying adaptation to a kinematic perturbation (Flanagan et al., 1999; Krakauer et al, 1999). While we should remain cautious in making direct comparisons of the current results to those obtained using a dynamic perturbation, we suggest here that learning a visuomotor rotation task may in fact occur in a plan-based or aim-based frame once we unconfound all of the reference frames.

The results here also predict that one could learn two different sensorimotor mappings that overlap either target location or hand paths, provided that the aiming direction is sufficiently far apart to prevent catastrophic interference. Indeed, a recent study (Hirashima and Nozaki, 2012) found that participants were able to counter opposing perturbations despite sharing the same direction of hand motion, although planned movement and target directions were conflated. Future work that dissociates planned aiming direction from target location, as well as hand location, is needed to determine the extent to which the pattern of generalization is dependent on trial-by-trial fluctuations of aim.

An interesting phenomenon observed in our data is the lack of symmetry in the between-subject generalization curve. We found a sharper decline in generalization in the direction opposite to the perturbation. We interpret these results by considering that participants made hundreds of consecutive reaches to the same location. Several previous studies have shown that movements following a series of repeated movements are biased in the direction of repeated motion, a process known as use-dependent plasticity (Diedrichsen et al., 2010; Huang et al., 2011). Here, the repeated hand motion is at 45° counterclockwise of the target. Catch trials that were collected at angles counterclockwise of the 45° reach direction were biased in the clockwise direction, appearing as reduced generalization. Conversely, catch trial measurements clockwise relative to the 45° repeated motion were biased in the counterclockwise direction, thus appearing as greater generalization. Both of these results can be explained by use-dependent plasticity driving the reach closer to the repeated direction of movement.

Our data have implications for the interpretation of computational models of motor control. Because we find that generalization is centered around the aiming location, the representation of learning is centered about a more abstract level for each individual and, importantly, does not appear to be tied to any measurable task dimension (e.g., hand path or target location). Consequently, attempting to extract motor learning principles from a particular generalization function may prove difficult if individuals have idiosyncratic and/or faulty strategies (Taylor et al., 2014). Such idiosyncrasy may account for the vast range of patterns of generalization in the literature, which were previously thought to be a reflection of the influence of mixed reference frames (Brayanov et al., 2012; Berniker et al., 2013), environmental statistics (Thoroughman and Taylor, 2005), and context dependency (Taylor and Ivry, 2013) on generalization. This is especially challenging for neural network models, which have sought to infer the properties of the underlying neural representation from the pattern of generalization but have relied on centering learning about target position (Thoroughman and Shadmehr, 2000; Donchin et al., 2003; Thoroughman and Taylor, 2005; Tanaka et al., 2009; Pearson et al., 2010; Brayanov et al., 2012; Taylor et al., 2013). Our findings here suggest that these models would need to update the network based on a more cognitive representation of where participants aim to move. Because aiming can change rapidly from trial-to-trial, the representation would also be rapidly changing and highly dynamic both within an individual and between individuals, making it exceedingly difficult to infer a consistent underlying representation using the neural network approach. Gaining a better understanding of the cognitive influences on motor adaptation will be crucial to the appropriate implementation of computational and neural network models used to explain the behaviorally observed generalization patterns.

In conclusion, we have shown that visuomotor adaptation generalizes around a cognitive construct of how we think we move. This finding reconciles a series of seemingly contradictory reports on how the nervous system generalizes adapted motor patterns and further underscores the importance of cognitive contributions to motor adaptation. There is obvious interplay between the cognitive and implicit processes involved in motor adaptation (Mazzoni and Krakauer, 2006; Taylor et al., 2014); here, we have demonstrated that the two are not merely engaged in a simple give-and-take relationship to achieve task goals, but rather the implicit sensorimotor recalibration that defines visuomotor adaptation is learned around the cognitive representation of the movement.

## Synthesis

The decision was a result of the Reviewing Editor Philippe Tobler and the peer reviewers coming together and discussing their recommendations until a consensus was reached. A fact-based synthesis statement explaining their decision and outlining what is needed to prepare a revision is listed below. The following reviewer(s) agreed to reveal their identity: Luc Selen

Dear Dr. Bastian,

Your manuscript has now been reviewed by two experts in the field. While they found merit, they also provide suggestions for how to improve clarity and impact. I agree with them. If you feel that you can address their comments, we would consider a revision.

Best regards,

Philippe Tobler

Synopsis

This study presents some interesting results on implicit visuomotor learning, although it's a bit hard to follow exactly what is going on in a first read. The main result seems to be that implicit learning calculated as the difference between explicit movement aim and the actual movement only agrees with the implicit learning measured in a catch trial if that catch trial is directed at the explicit movement target.

General comments

1)The big assumption in this paper is that subjects can cognitively 'ignore' the aiming strategy they were forced to adopt in the learning trials during the probing trials. Can this assumption be put on firmer ground? The subjects certainly change something, but what exactly?

2)Relatedly, there is a clear and systematic observation, but little suggestion of how to model this. All the discussion is about reinterpreting old data in terms of this new finding. But would the brain always do this? And how is this finding special for the experiments in which the subject is forced to indicate the aim location or does it also hold for 'normal' visuomotor learning and the formation of a generalization curve. Perhaps one lead could come from figure 5, which shows the 'generalization' (although this is not really the generalization curve, but the correspondence curve between the implicit component derived from subtracting the reach direction from the indicated aim location (i.e., the trick introduced by Taylor) and the supposedly measured implicit component) across subjects. While it is important to show the distribution of aim locations, hand positions and cursor locations, this presentation has the suggestion of a model but also other models are conceivable.

3)It needs to be made clearer (e.g. in the title too) that the study relates to visuomotor learning and not to motor learning in general. Accordingly, in the introduction it would be better to give examples of visuomotor rotations rather than general motor learning

4)Overall the explanation of the methods needs to be improved and simplified. In particular, how explicit and implicit learning are calculated and also how catch trials are used to assess implicit learning need more explanation and discussion. The latter in particular is a pivotal issue on which the claims made in the manuscript strongly depend.

5)Relatedly, the statement "Importantly, here we also included a direct measurement of implicit learning." needs to be explained better. Do the catch trials really provide a direct and unique measure of implicit learning? Aren't they also affected by explicit aiming too and the landmarks are simply not available?

6)It would be helpful to improve the figures. More specifically, Figs 1 and 2 are important to the paper and improving the clarity of these would be helpful. Fig 1 would benefit from being drawn larger and also all of its details should be explained in the text.

7)It is unclear why the authors have not built a within-subject generalization curve. This would massively strengthen their claims and would make figures 6 and 7 completely unnecessary. (Figure 6 does not seem to add to the story. It shows that the aim location results in better generalization than the target location within subjects. This is a replication of what they show in figure 2, but here it is across subjects so it could be combined with figure 2. Figure 7 is essentially the same story, now based on all catch and aftereffect trials. It can be removed.)

8)There is some slightly strange phrasing (e.g. in the abstract). Also the writing style sometimes seems a bit over the top and exaggerated (see detailed comments).

Detailed comments

1)At the start of the methods section, it would be good to introduce the different target angles used for the catch trials, which forms the basis for claims of generalization. This is only mentioned later.

2)"Human motor learning is most useful if it generalizes beyond the trained task." ⇒ remove "most"

3)"Here, we introduce a new idea about how human motor learning generalizes." ⇒ "new results regarding how human visuomotor learning generalizes."

4)"Feedback of their unseen hand was displayed as a cursor that was rotated to induce learning. " ⇒ rephrase

5)"Generalization is one of the most important features of human motor learning," ⇒ Generalization is one important feature of human motor learning,

6)"Here, we provide a new perspective on motor learning generalization..." ⇒ "Here, we provide a new perspective on motor learning generalization of visuomotor rotations...

7)"These mechanisms are often studied using a visuomotor reaching task..." The authors may want to acknowledge that although visuomotor tasks are often used to investigate these issues, studies involving dynamic learning tasks are also used. Also, the authors do not discuss their finding in relation to force field learning. This is a different kind of learning, but at least they should mention Gonzalez Castro et al., 2011 (Plos Comput. Biol.) about updating internal models based on target movement or executed movement. Maybe from that model they can sketch an avenue of how to interpret the current findings.

8)"It has been difficult to answer this question in previous studies because participants feel that their hand is moving with the cursor by the end of adaptation, when it is in fact moving in a rotated direction" ⇒ "It has been difficult to answer this question in previous studies because participants feel that their hand is moving with the cursor by the end of adaptation, although by this point it is in fact moving in a rotated direction"

9)"Here we show that, in a situation where participants are asked to report their aiming location, implicit motor learning generalizes around the aiming location (i.e. where they intended to move) rather than the target location (i.e. where the cursor moved)." ⇒ -> so by the end of the learning task, they aim for the target and generalization is then around target?

10)"In past studies, the implicit component of visuomotor adaptation was abstracted by subtracting the reported aim from the reach angle (Figure 1A; Taylor et al., 2014)." ⇒ It would be helpful to illustrate this in the manuscript and not just refer to a fig. and a reference.

11)"These single catch trials had the same format as the aftereffect measurement trials (purple block) in that endpoint feedback, landmarks, and rotation were removed." ⇒ If there is no feedback why is it necessary to remove rotation?

12)"We first tested a group of participants that aimed at the trained target (i.e. 0{degree sign} location) for all catch and aftereffect trials-named 'Target' group here. We noticed an interesting discrepancy between the calculated implicit component and the directly measured catch trial magnitudes (Figure 2A; see the difference between pink and green curves). Given that adaptation is thought to generalize maximally around the target location (Krakauer et al., 2000), why did the calculated implicit component and catch trial magnitudes when tested at 0{degree sign} not match?" ⇒ This discussion of results appears in the methods section whereas the results section would be more appropriate.
